# Sequence Diversity of Tp1 and Tp2 Antigens and Population Genetic Analysis of *Theileria parva* in Unvaccinated Cattle in Zambia’s Chongwe and Chisamba Districts

**DOI:** 10.3390/pathogens11020114

**Published:** 2022-01-19

**Authors:** Walter Muleya, David Kalenzi Atuhaire, Zachariah Mupila, Victor Mbao, Purity Mayembe, Sydney Kalenga, Paul Fandamu, Boniface Namangala, Jeremy Salt, Antony Jim Musoke

**Affiliations:** 1 Department of Biomedical Sciences, School of Veterinary Medicine, University of Zambia, Lusaka 10101, Zambia; muleyawalter@gmail.com (W.M.); mupilatambwe@gmail.com (Z.M.); 2 Centre for Ticks and Tick-Borne Diseases, Lilongwe, Malawi; 3 Eastern and Southern Africa Regional Office, International Development Research Centre, Nairobi 00200, Kenya; vmbao@idrc.ca; 4 Department of Veterinary Services, Ministry of Fisheries and Livestock, Lusaka 50060, Zambia; puritymayembe@yahoo.com (P.M.); kasidn@gmail.com (S.K.); pfandamu@gmail.com (P.F.); 5 Department of Paraclinical Studies, School of Veterinary Medicine, University of Zambia, Lusaka 10101, Zambia; b.namangala@unza.zm; 6 Global Alliance for Livestock Veterinary Medicines, Pentlands Science Park, Bush Loan, Penicuik EH26 0PZ, UK; jeremysalt99@gmail.com; 7 LMK Medical Laboratories and Consultancies, Kampala P.O. Box 33686, Uganda; tomusoke@gmail.com

**Keywords:** *Theileria parva*, ECF immunization, sub-structuring, Tp1, Tp2, Zambia

## Abstract

East Coast Fever (ECF), caused by *Theileria parva*, is a major constraint to improved livestock keeping in east and central Africa, including Zambia. To understand the dynamics and determine the candidates for immunization in Zambia’s Chongwe and Chisamba districts, a combination of Tp1 and Tp2 gene sequencing and microsatellite analysis using nine markers was conducted from which an abundance of Muguga, Kiambu, Serengeti and Katete epitopes in the field samples was obtained. Phylogenetic analysis showed six (Tp1) and three (Tp2) clusters with an absence of geographical origin clustering. The majority of haplotypes were related to Muguga, Kiambu, Serengeti and Katete, and only a few were related to Chitongo. Both antigens showed purifying selection with an absence of positive selection sites. Furthermore, low to moderate genetic differentiation was observed among and within the populations, and when vaccine stocks were compared with field samples, Chongwe samples showed more similarity to Katete and less to Chitongo, while Chisamba samples showed similarity to both Katete and Chitongo and not to Muguga, Kiambu or Serengeti. We conclude that the use of Katete stock for immunization trials in both Chongwe and Chisamba districts might produce desirable protection against ECF.

## 1. Introduction

Theileriosis, a lymphoproliferative disease caused by a tick-borne hemoprotozoan parasite known as *Theileria parva* (Theiler, 1904) and transmitted by *Rhipicephalus appendiculatus* [1], is widely distributed in eastern, central and southern Africa. The manifestation of the disease is dependent on the cattle (*Bos taurus*) breed, with exotic breeds suffering more devastating effects than local indigenous zebu breeds [2,3]. In Zambia, East Coast Fever (ECF) traditionally occurs as a mild and severe form of theileriosis in the eastern province and southern provinces, respectively. However, with the recent spread of theileriosis to different parts of the country, mixed forms of the disease have been observed and, due to the lack of characterization of the strains prevailing in these areas, no countrywide immunization program has been implemented. Cattle are a major source of economic strength for the local communities in Zambia, and mortality due to theileriosis is a huge constraint to the development of the livestock industry. Theileriosis was previously reported to cause approximately 10,000 deaths per annum [4], but currently, this number could be higher. Apart from cattle and buffaloes (*Syncerus caffer*), *Theileria* spp. have also been identified in African sable antelopes (*Hippotragus nige*) in South Africa [5] and Botswana [6].

Molecular techniques have been used to identify the species and strains of *T. parva* circulating in different areas [7,8,9,10]. Further, these techniques have been able to distinguish buffalo-derived from cattle-derived *T. parva* stocks [11] and have shown that buffalo-derived strains have a higher diversity than the cattle-derived stocks [12,13,14,15]. In addition, several studies have described *T. parva* antigens and epitopes from immunized cattle [16,17,18,19]. Among these are Tp1 and Tp2, which have been extensively studied and reported to represent the immunodominant targets recognized by the CD8^+^ T-cells in cattle with MHC class I haplotypes [20]. Tp1 and Tp2 antigens have also exhibited extensive polymorphism in their sequences as well as epitope regions [21]. They have been utilized to determine the parasite populations prevailing in a particular area and how similar these parasites are to the common vaccine stocks [8,10,22]. In addition to Tp1 and Tp2, some studies have incorporated microsatellite analysis to genotype field populations followed by field evaluations of vaccine stocks that are most similar to field populations with very promising results for the implementation of immunization programs [8]. This approach has been used because microsatellite analysis is more informative and provides better genetic discrimination than sequence analysis.

In Zambia, ECF is currently controlled using an integrated system of livestock movement control, chemotherapy, tick vector control via acaricides and immunization with the infection and treatment method (ITM) using the cattle-derived Katete (Eastern province) [23,24] and Chitongo (Southern province) [25] single stocks, respectively. In order to completely protect cattle in Zambia against theileriosis, effective immunization programs beyond the current two provinces are required. However, prior to the implementation of these programs, the assessment of parasite populations in these respective areas is essential. Chongwe and Chisamba districts located in Zambia’s Lusaka and Central provinces, respectively, have been considered as theileriosis-free areas in the past. As such, no immunization programs have been implemented despite the possible presence of theileriosis.

Thus, this study was conducted to determine whether *T. parva* is present in Zambia’s Chongwe and Chisamba districts and compare it to known immunizing stocks such as the Muguga, Kiambu, Serengeti, Chitongo and Katete by analyzing the sequence diversity of Tp1 and Tp2 antigens. Furthermore, the study aimed at determining the extent of gene flow and the population structure of *T. parva* in Chongwe and Chisamba districts of Zambia using a panel of nine satellite markers. The information obtained in this study has the potential to aid in the introduction of immunization trials in Chongwe and Chisamba districts. To the best of our knowledge, this is the first study that has incorporated sequence diversity of *T. parva* antigens and micro- and mini-satellite analysis in Zambia.

## 2. Results

### 2.1. Theileria parva PCR Screening

*Theileria parva* genomic DNA was detected in all the samples from Chongwe (*n* = 130) and Chisamba (*n* = 29) districts, respectively, using p104 gene PCR.

### 2.2. Sequence Diversity, Phylogenetic and Similarity Analyses of the Tp1 Gene Locus

The 403 bp of the Tp1 gene of *T. parva* was successfully sequenced from 97/130 and 27/29 samples from Chongwe and Chisamba districts, respectively. Amino acid sequences were translated from the obtained nucleotide sequences and then compared to the Muguga, Kiambu, Serengeti, Katete and Chitongo amino acid reference sequences. The epitope VGYPKVKEEML present in Muguga, Kiambu and Serengeti [21] was identified in 82/97 and 20/27 samples from Chongwe and Chisamba districts, respectively. Katete vaccine stock shared the same epitope with Muguga, Kiambu and Serengeti. The epitope VGYPKVKEEII present in Chitongo [21] was only identified in 10/97 and 6/27 samples from Chongwe and Chisamba districts, respectively. The remaining samples from Chongwe (*n* = 5) possessed epitopes VGYPKV**R**EEML (*n* = 1) and VGYPKVKEEM**I** (*n* = 4). With regards to Chisamba, only one (1) sample possessed an epitope (VGYPKVKEEM**I**) that was different from Muguga, Kiambu, Serengeti, Chitongo and Katete. Therefore, the epitope, common to Muguga, Kiambu, Serengeti and Katete, is the most abundant epitope in both districts, whereas the Chitongo epitope was detected in less than 10% of the samples.

**Figure 1:**
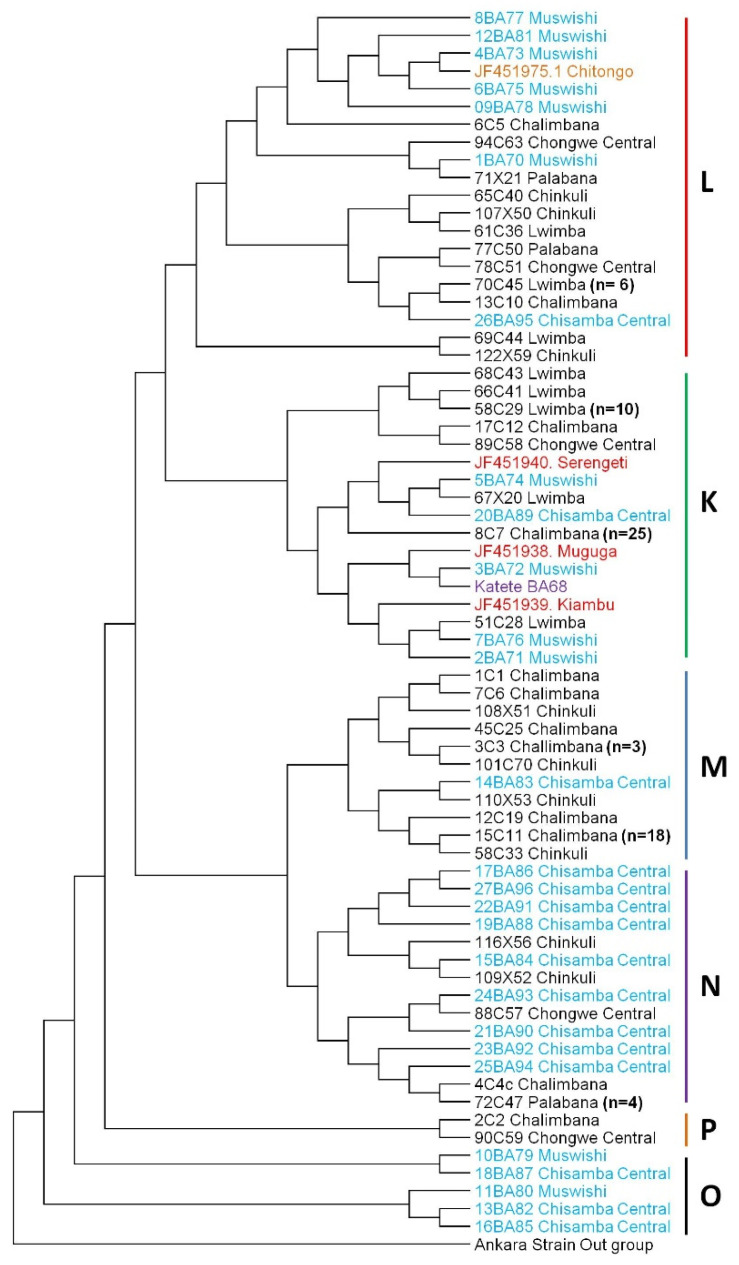
*Theileria parva* CTL Tp1 neighbour-joining phylogenetic tree. The phylogenetic tree was constructed from 432 bp nucleotide sequences using MEGA ver. 6 with a confidence interval of 1000 bootstrap replicates. Sequences that shared 100% homology (Supplementary Table S1) are represented by a single sequence on the phylogenetic tree. Samples from Chisamba district are given in blue while those from Chongwe district are given in black. Muguga cocktail (Muguga, Kiambu and Serengeti), Katete and Chitongo vaccine isolates are given in, red, purple and brown, respectively.

Phylogenetic analysis of the samples under study showed that 41/97 sequences from Chongwe district formed a cluster with the Muguga, Kiambu, Serengeti and Katete vaccine strains (cluster K) (Figure 1). The remaining sequences from Chongwe formed a cluster with Chitongo (cluster L, *n* = 15/97) and could also be found in clusters M (*n* = 29/97), P (*n* = 2/97) and N (*n* = 8/97) that were separate but related to the vaccine clusters K and L (Figure 1). There was no evidence of clustering according to the veterinary camp of origin of the samples within Chongwe district. With regards to sequences from Chisamba district, 5/27 clustered with Muguga, Kiambu, Serengeti and Katete vaccine strains in cluster K and 7/27 with Chitongo vaccine in cluster L. The remaining 15/27 sequences clustered in M (*n* = 1), N (*n* = 9) and O (*n* = 5) (Figure 1). The majority of sequences from Chisamba district formed a separate cluster N away from Muguga, Kiambu, Serengeti, Katete and Chitongo vaccine strains. Overall, phylogenetic analysis revealed that 41/97 sequences from Chongwe were similar to Muguga, Kiambu, Serengeti and Katete strains while only 15/97 were similar to Chitongo, thus Muguga cocktail and Katete strains were more represented in Chongwe district (Figure 1). With regards to Chisamba district, phylogenetic analysis showed the clustering of field samples with Muguga, Kiambu, Serengeti, Katete and Chitongo vaccine strains. However, the majority of sequences did not cluster with the vaccine groups (Figure 1). Furthermore, a DNA polymorphism and dN/dS mean ratio of π = 0.865% and 0.97 with one positive selection site and zero negative selection sites, respectively, was observed. Analysis of molecular variance (AMOVA) of both the Chongwe and Chisamba populations revealed that 92% of the genetic variation observed was within the population while 8% was among the population. Treating Chongwe and Chisamba populations as separate populations further showed that 97% and 90% of the variation was within the population while 3% and 10% were among the population for Chongwe and Chisamba, respectively.

**Figure 2:**
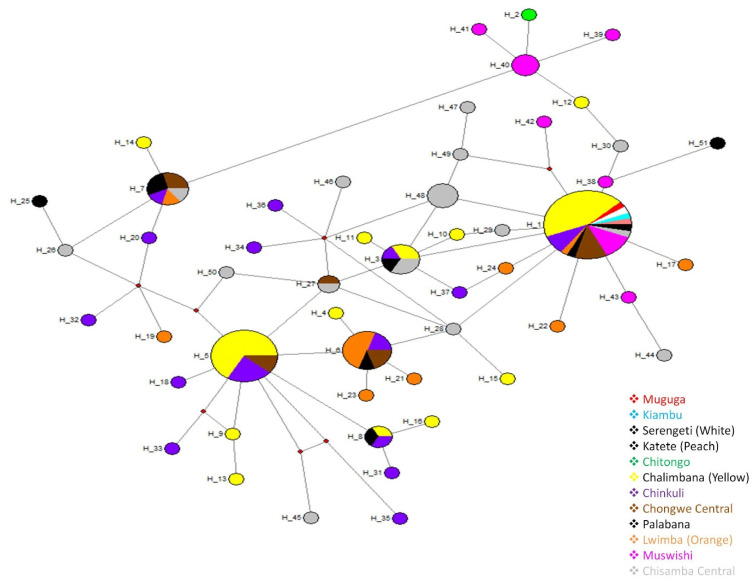
*Theileria parva* Tp1 gene median-joining network (MJ) constructed using Network 4.5 based on the Tp1 sequences. Sample origins are given by color codes, and the size of the circles represent the haplotype frequency. The MJ network shows a star-like radiation pattern.

The similarity of the haplotypes between Muguga, Kiambu, Serengeti, Chitongo, Katete and the field samples was analyzed using network ver. 4.5 [26]. The MJ network showed that the most abundant haplotypes were H1(*n* = 34) and H5 (*n* = 18), and from these, several haplotypes were seen radiating (Figure 2).

H1 comprised of Muguga, Kiambu, Serengeti, Katete and samples from Chongwe (*n* = 25) and Chisamba (*n* = 5), while H5 exclusively comprised of samples from Chongwe (*n* = 18). H48 (*n* = 4) and H40 (*n* = 3) comprised only samples from Chisamba and were linked to H1 and H2 (Chitongo), respectively. With the exception of haplotypes H1, H3, H5, H6, H7, H8 and H27, samples from Chongwe and Chisamba did not share any other haplotypes (Figure 2). Overall, the network link showed that the most common haplotype in Chongwe district was similar to the haplotype expressed by Muguga, Kiambu, Serengeti and Katete isolates, while in Chisamba, a few independent haplotypes as well as those related to Chitongo and similar to or closely linked to Muguga, Kiambu, Serengeti and Katete, were present. A star-like radiating pattern indicative of population expansion was also observed (Figure 2).

### 2.3. Sequence Diversity, Phylogenetic and Similarity Analyses of Tp2 Gene Locus

The 496 bp of the Tp2 gene of *T. parva* was sequenced from the 128/130 samples from Chongwe and 14/29 samples from Chisamba district, respectively. Amino acid sequences were translated from the obtained nucleotide sequences and compared to the amino acid sequence of the Muguga reference sequence (XP_765583), from which six epitopes have so far been identified, namely; CTL 1 (SHEELKKLGML), CTL 2 (DGFDRDALF), CTL 3 (KSSHGMGKVGK), CTL 4 (FAQSLVCVL), CTL 5 (QSLVCVLMK) and CTL 6 (KTSIPNPCKW) [21]. Within Chongwe district, across epitopes 1–6, the epitopes present in the Muguga, Kiambu, Serengeti and Katete were identified in 89, 99, 98, 101, 100 and 109 samples, while the Chitongo epitopes were only identified in 13, 5, 7, 7, 6 and 2 samples, respectively. A total of 26, 24, 23, 20, 22 and 17 samples possessed epitopes that were different from those present for either Muguga, Kiambu, Serengeti, Katete or Chitongo. From Chisamba district, 4, 6, 6, 6, 6 and 7 samples, respectively, possessed 100% similar epitopes across all six epitope regions with components of the Muguga, Kiambu, Serengeti and Katete while 5, 6, 4, 4, 4 and 6 samples had 100% similarity with Chitongo across all the six epitopes. The number of samples that had a different epitope from that found in the Muguga, Kiambu, Serengeti, Katete and Chitongo strains was 5, 2, 4, 4, 4 and 1, respectively, on each of the six epitopes. Overall, the majority of epitopes identified from both Chongwe and Chisamba districts were similar to epitopes present in the Muguga, Kiambu, Serengeti and Katete isolates. The total number of epitopes identified in this study that were different from Muguga, Kiambu, Serengeti, Chitongo and Katete, indicative of different parasite strains, are provided in Table 1.

**Figure 3:**
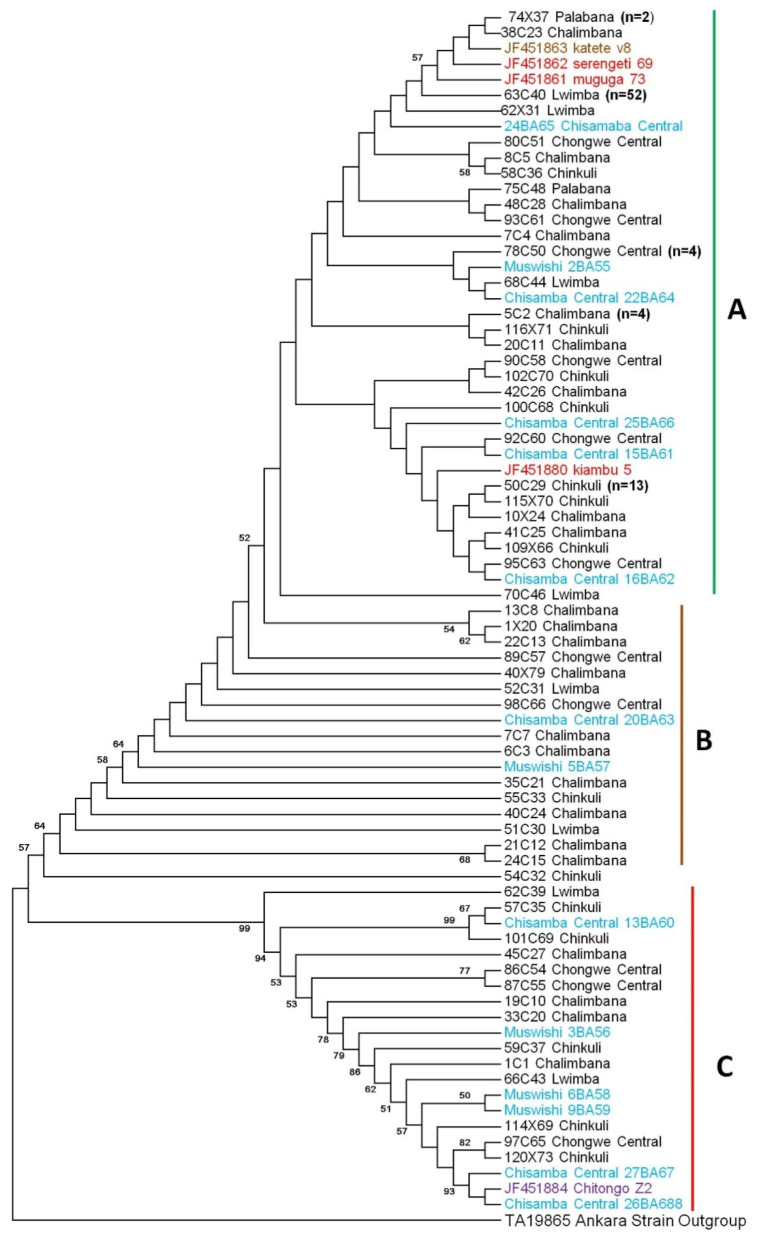
*Theileria parva* CTL Tp2 neighbour-joining phylogenetic tree. MEGA ver. 6 was used to construct the phylogenetic tree based on the 531 bp Tp2 nucleotide sequences with 1000 bootstrap replicates as confidence level. Samples from Chongwe and Chisamba districts are given in black and blue, respectively. The Katete, Chitongo and Muguga, Kiambu and Serengeti vaccine isolates are given in brown, purple and red, respectively. For sequences sharing 100% homology, only a single sequence was used to represent the group (Supplementary Table S1).

Phylogenetic analysis of the Tp2 nucleotide sequences (Figure 3) from Chongwe revealed that the majority of sequences (*n* = 98/128) clustered with Muguga, Kiambu, Serengeti and Katete vaccine strains (cluster A) while only 14/128 sequences clustered with the Chitongo vaccine strain (cluster C) (Figure 3).

The rest of the sequences (*n* = 16) were loosely arranged in cluster B but related to clusters A and C. With regards to Chisamba district, 6/14 sequences clustered with Muguga, Kiambu, Serengeti and Katete vaccine strains in cluster A while a similar number of sequences (*n* = 6) clustered with Chitongo in cluster C (Figure 3). The rest of the Chisamba samples (*n* = 2) clustered with Chongwe samples that were loosely arranged in cluster B and related to clusters A and C. Overall, the majority of sequences from Chongwe district were more similar to Muguga, Kiambu, Serengeti and Katete strains as compared to Chitongo, and only a few samples (*n* = 16) did not share a cluster with the vaccine strains. On the other hand, within the samples from Chisamba district, an equal representation of Muguga, Kiambu, Serengeti and Katete strains as well as Chitongo strain was observed. Furthermore, there was no evidence of clustering according to the veterinary camp of origin (Figure 3). The DNA polymorphism calculated was π = 7.3%, and the mean ratio of dN/dS was 0.634 with 26 negative selection sites and 6 positive selection sites. Analysis of molecular variance showed that 1% of the variation was among the populations while 99% was within the population when Chongwe and Chisamba were treated as a single population. When Chongwe and Chisamba were treated as separate populations, each district showed 0% variation among the populations and 100% variation within the populations.

**Figure 4:**
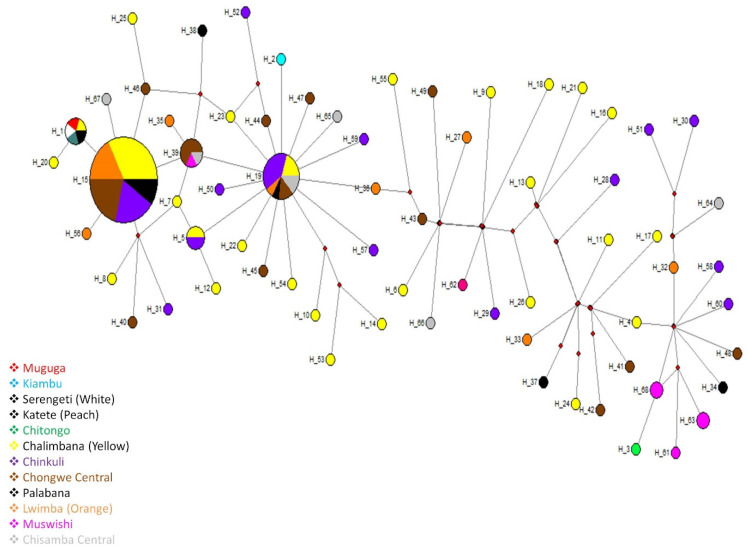
*Theileria parva* Tp2 gene median-joining network based on Tp2 sequences from Chongwe and Chisamba districts. The MJ network shows a star-like radiation pattern, and the sizes of the circles correspond to the frequency of haplotypes. The sample origins are given by respective color codes. The MJ network was constructed using Network ver. 4.5.

Haplotype similarity among Muguga, Kiambu, Serengeti, Katete and Chitongo vaccine strains and the field samples from Chongwe and Chisamba districts (Figure 4) revealed that the most abundant haplotype was H15 (*n* = 52) followed by H19 (*n* = 15).

H15 was exclusively comprised of Chongwe samples, while H19 comprised of both Chongwe (*n* = 13) and Chisamba (*n* = 2) samples. H18 and H14 were indirectly linked via H39 comprising of Chongwe (*n* = 4) and Chisamba (*n* = 2) samples. Several haplotypes such as H1(*n* = 5) comprising of Muguga (*n* = 1), Serengeti (*n* = 1), Katete (*n* = 1) and samples from Chongwe (*n* = 2) were seen radiating from and forming a star-like pattern from H15 which also formed the anchor of the network. In the same manner that H15 was directly linked to H1, H19 was also directly linked to H2, which comprised of the Kiambu vaccine isolate. However, H3 (Chitongo isolate) had no direct link to any of the Chongwe samples but was only directly linked to H68 comprising of Muswishi samples from Chisamba (*n* = 2), which was also linked to H61 and H63 (both from the Muswishi area in Chisamba) via a median vector. With the exception of H5, no other haplotypes were shared among the samples from both Chongwe and Chisamba districts. Overall, the MJ network revealed that the most abundant haplotype in Chongwe was directly linked to but different from Muguga, Serengeti, Katete and Kiambu, while for Chisamba district, the most abundant haplotype was directly linked to Chitongo but was different. Collectively, population expansion was also observed on the MJ network constructed from samples from Chongwe and Chisamba districts (Figure 4).

### 2.4. Microsatellite Marker and Similarity Analysis

In order to genotype field samples from Chongwe and Chisamba districts and compare them with Muguga, Kiambu, Serengeti, Chitongo and Katete vaccine isolates, a panel of nine satellite markers spanning the four chromosomes of *T. parva* was used. The most polymorphic marker for Chongwe population was MS14, identifying 55 alleles, while the least polymorphic was MS2 identifying 13 alleles (Table 2).

**Figure 5:**
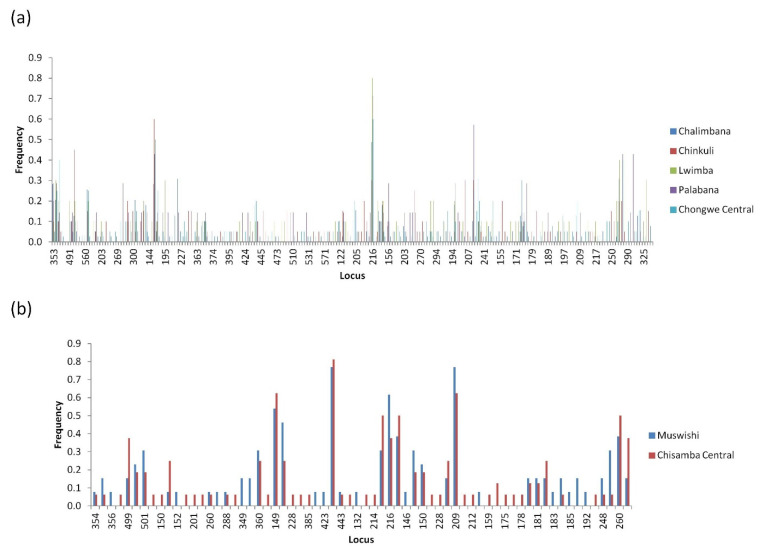

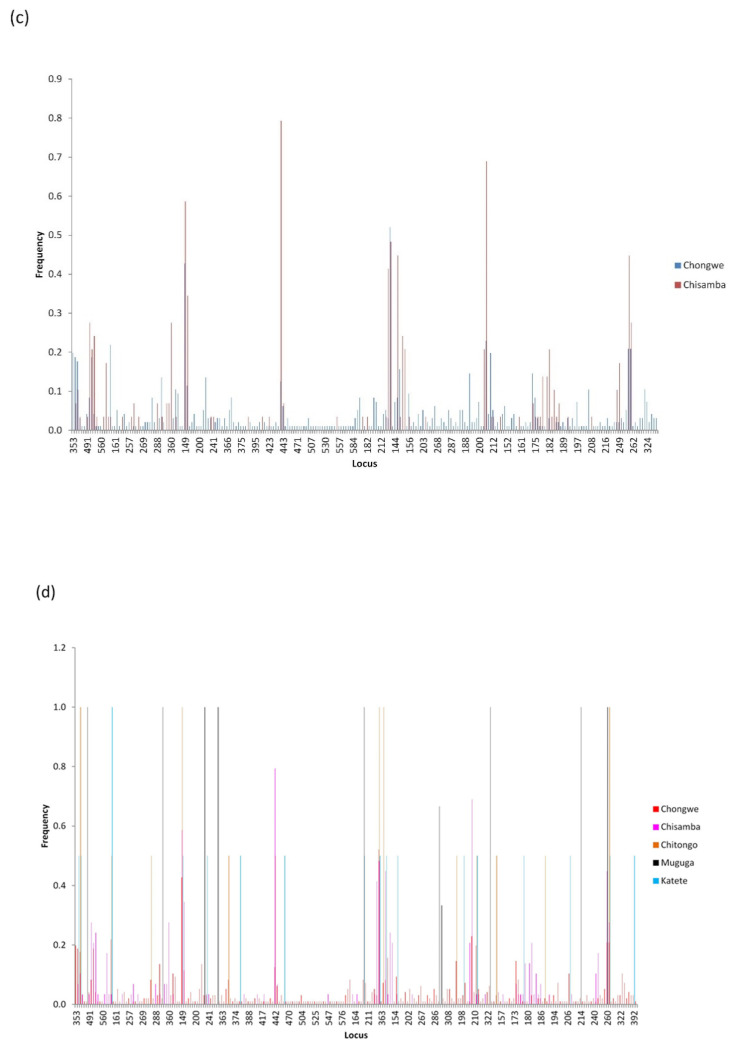
Allele frequencies of field samples from Chongwe and Chisamba districts. (**a**) Allele frequencies from Chongwe district. (**b**) Allele frequencies of field samples from Chisamba district. (**c**) Allele frequencies generated from field samples when samples from Chongwe and Chisamba districts were treated as a single population. (**d**) Allele frequencies from field samples from Chongwe and Chisamba district together with the Muguga cocktail (MC), Katete and Chitongo vaccine isolates. Shared and unique alleles are represented in (**a**–**d**). The predominant alleles calculated as proportions of the total of each marker are presented as histograms generated from the multi-locus genotype.

In Chisamba population, the most polymorphic markers were MS7 and MS33, each identifying 14 alleles, while the least polymorphic were MS9 and MS39, each identifying 4 alleles. Overall, the highest mean number of alleles identified was 13.2 (MS14), and the least number identified was 4.6 (MS15). High gene diversities were observed on all loci in both Chongwe and Chisamba populations except for markers MS14 and MS25, which recorded low diversities in Chisamba district (Table 2). Overall, across all loci, the highest gene diversity was 0.77 (MS33), and the least was 0.464 (MS15) (Table 2). With respect to allele frequencies (Figure 5), treating Chongwe and Chisamba district populations as single populations revealed 51 and 28 shared and 245 and 38 unique alleles, respectively (Figure 5a,b). The higher proportion of unique alleles as compared to shared alleles was indicative of genetic differentiation.

**Figure 6:**
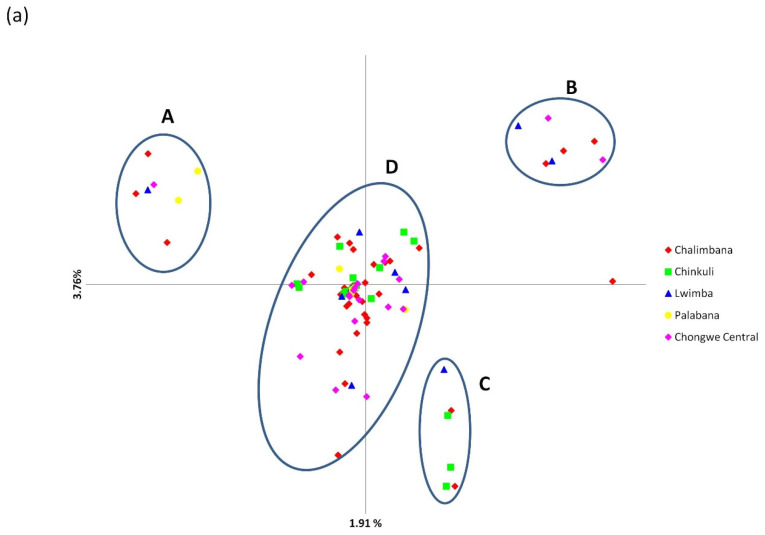

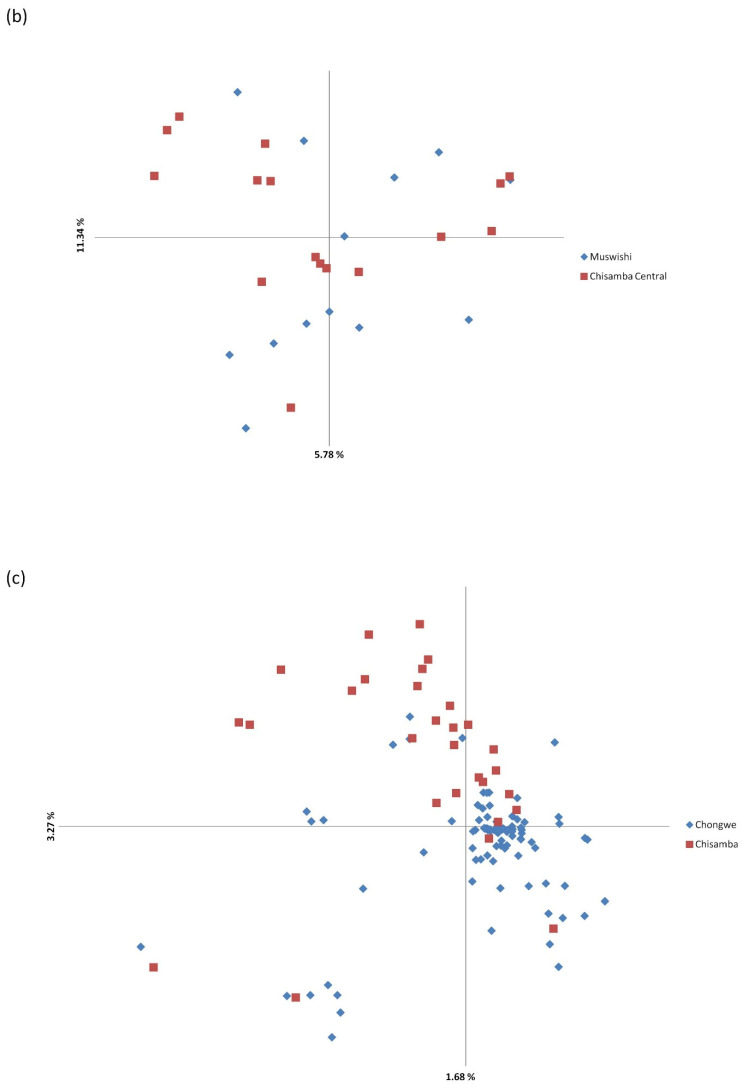

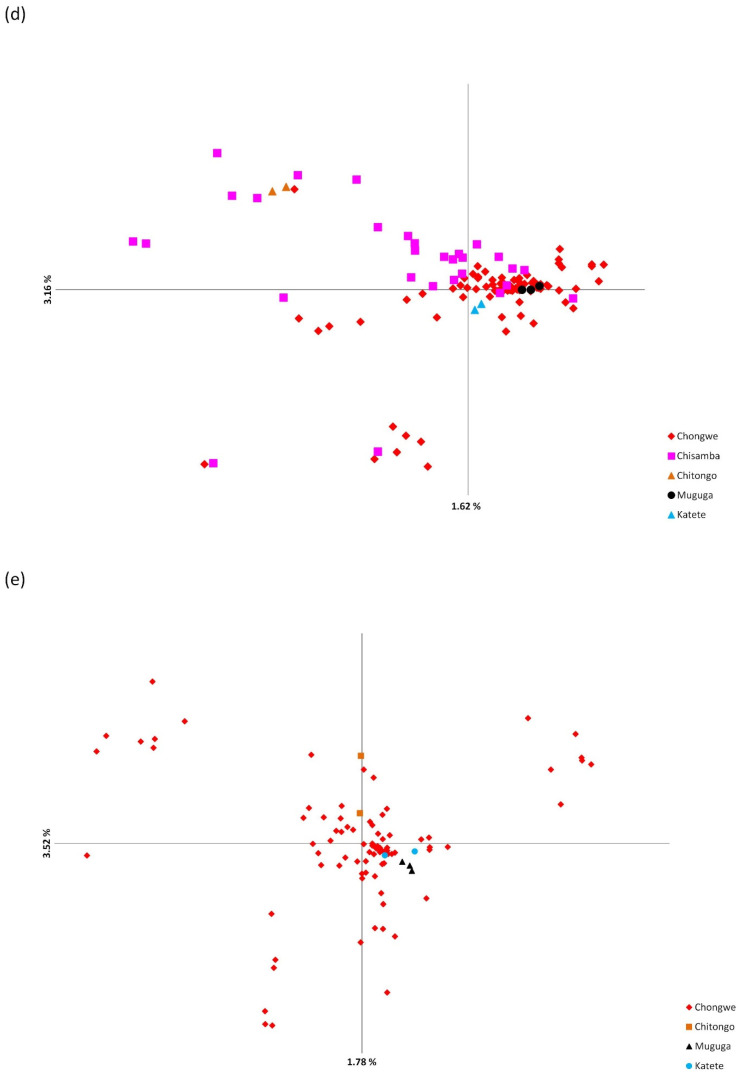

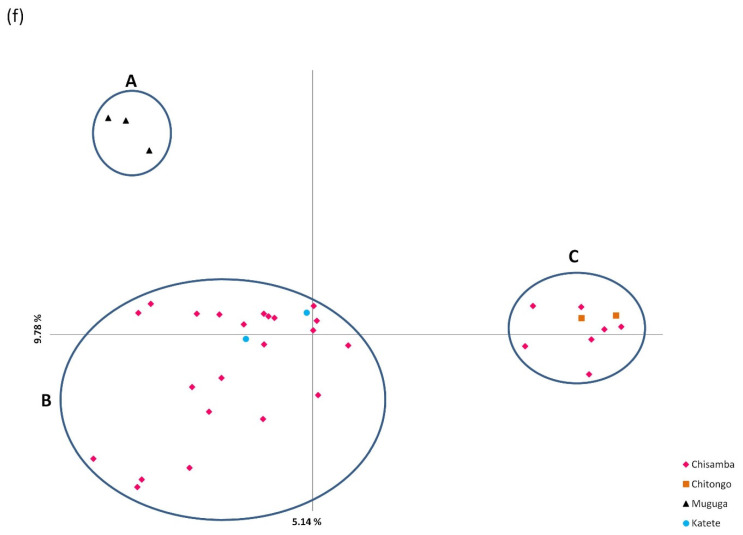
Principal component analysis (PCA) of *Theileria parva* from field populations and vaccine isolates constructed from multi-locus genotype data. The variation in proportion in the population data set is given on each axis. (**a**) Chongwe district field samples from different veterinary camps occupying all quadrants and divided into four clusters irrespective of sample origin. (**b**) Field samples from Chisamba central and Muswishi veterinary camps in Chisamba district occupying all quadrants of the PCA plot without clustering according to origin. Moderate genetic differentiation can be observed. (**c**) PCA plot indicating low genetic sub-structuring between the Chongwe and Chisamba district field populations. (**d**) PCA plot of the field samples together with the Muguga cocktail, Katete and Chitongo vaccine isolates showing close relatedness of the Muguga and Katete isolates to the field samples. (**e**) PCA of Chongwe and vaccine isolates showing close clustering of Katete isolate with field samples. Both Muguga cocktail and Chitongo isolates show slight differentiation from the field samples from Chongwe district. (**f**) Field samples from Chisamba district, together with the Muguga cocktail, Katete and Chitongo vaccine isolates divided into clusters. The Muguga cocktail vaccine isolate forms a distant and independent cluster while the majority of field samples cluster with Katete isolate and the minority with Chitongo isolate.

Principal component analysis (PCA) (Figure 6) of both populations showed the distribution of samples from different veterinary camps across the PCA plot; that is, samples from respective veterinary camps did not form separate clusters (Figure 6a,b).

For Chongwe (Figure 6a), four distinct clusters were also observed namely A, B, C and D. The samples originated from Chalimbana, Chongwe central and Lwimba for cluster A, Chalimbana, Lwimba, Chongwe central and Palabana for cluster B, Chinkuli, Chalimbana and Lwimba for cluster C and finally Lwimba, Chongwe Central, Chikuli, Palabana and Chalimbana for cluster D (Figure 6a). When Chisamba and Chongwe populations were treated as a single population, a total of 41 shared and 209 (Chongwe = 185, Chisamba = 24) unique alleles were obtained (Figure 5c). The high number of unique alleles was suggestive of population differentiation/sub-structuring (Figure 5c). This was further investigated using PCA (Figure 6c), which showed that most of the field samples from Chongwe district occupied the lower left and right quadrants while those from Chisamba district occupied the upper left and right quadrants indicative of population sub-structuring. When all populations, including the vaccine stocks, were treated as a single population, 42 shared alleles and 242 unique alleles were observed (Figure 5d). For the sake of simplicity, we shall refer to Muguga, Kiambu and Serengeti collectively as the Muguga cocktail (MC) population in reference to all population genetic analyses performed in this study. Chongwe and Chisamba populations did not share any alleles with MC or Katete vaccine populations. However, both populations shared a total of seven alleles with the Chitongo vaccine population (Figure 5d). The population differentiation and level of similarity demonstrated by allele frequency analysis among the populations were further assessed using PCA (Figure 6d), which showed close clustering of MC and Katete populations with Chongwe populations, while the Chitongo vaccine population showed similarity with only a few samples from Chisamba district. In order to observe the patterns on PCA in finer detail, PCA of Chongwe and Chisamba populations with the respective vaccines were constructed (Figure 6e,f). The PCA for Chongwe, MC, Katete and Chitongo populations (Figure 6e) showed clustering of field samples and vaccine isolates along the middle portion of the PCA. MC and Chitongo showed some extent of sub-structuring, while Katete showed close clustering with field sample populations indicating close similarity to the field samples. With respect to Chisamba (Figure 6f), PCA showed three clusters. Cluster A, occupying the top-left quadrant comprised of the MC population only, cluster B occupying the middle part and the left upper and lower quadrants comprised of Chisamba and Katete populations and lastly, cluster C, occupying the far right upper and lower quadrants, comprised of Chisamba and Chitongo populations. The sub-structuring patterns obtained from the PCAs for Chongwe (Figure 6a,e) and Chisamba (Figure 6b,f) indicate the possible presence of distinct parasite populations circulating within these districts.

### 2.5. Population Differentiation and Genetic Analysis

Across the different populations, similar and high levels of the estimated heterozygosity were observed except for the Chisamba population, which showed a moderate level of heterozygosity, possibly due to the reduced sample size (Table 3).

When both Chisamba and Chongwe populations were combined into a single population, an estimated heterozygosity of 0.8629 and a number of effective alleles of 7.426 were observed. The overall estimated heterozygosity and number of effective alleles for all the populations under study, including vaccines, were 0.8641 and 3.855, respectively (Table 3). The high estimated heterozygosities and number of alleles in each district is indicative of highly diverse populations. The extent of sub-structuring among the populations was further assessed using F statistics. When Chongwe, Chisamba, MC, Katete and Chitongo populations were taken as one population, an F_ST_ value of 0.1205, indicating moderate differentiation among the populations, was obtained (Table 3). When the populations from Chongwe and Chisamba were analyzed separately from other study populations, F_ST_ values of 0.036 and −0.012, indicative of low genetic differentiation, were obtained, respectively (Table 3). When Chongwe and Chisamba populations were analyzed as a single population, an F_ST_ value of 0.096, indicating moderate genetic differentiation, was obtained. Furthermore, to assess the level of panmixia within the Chongwe population, Chisamba population, Chongwe combined with Chisamba population and field populations combined with vaccine stocks, the levels of linkage equilibrium at all loci pairs were measured. Within the Chongwe population, a V_D_ value of 1.1059 and L value of 0.9673 (*p* < 0.01) with an index of association of 0.0260 indicating LD and non-panmixia were obtained (Table 3). Similarly, within the Chisamba population, a V_D_ value of 2.0003 and L value of 1.8916 (*p* < 0.01) with an index of association of 0.0233 indicating LD and non-panmixia were obtained (Table 3). When Chongwe and Chisamba populations were treated as a single population, a V_D_ value of 1.3044 and L value of 1.0690 with the index of association of 0.0373 indicating LD and non-panmixia were also obtained. Inserting the vaccine stocks into the field populations produced a V_D_ value of 1.3357, L value of 1.0518 and the index of association of 0.0421, indicating LD and non-panmixia (Table 3). Overall, population genetic analysis showed that *T. parva* populations in Chongwe and Chisamba districts were similar but diverse. In addition, these populations exhibited low to moderate genetic differentiation with an absence of random mating.

## 3. Discussion

Molecular approaches such as PCR and DNA sequencing have allowed for the detection of new pathogens in areas where they were previously thought to be non-existent. The sequencing of *T. parva* CTL antigens has also provided a means for analyzing the diversity of *T. parva* in endemic areas [8,9,10,21,22]. In Zambia, the communities of Chongwe and Chisamba rely on livestock keeping as a source of livelihood, and the presence of theileriosis is a negative attribute to the growth and improvement of the livestock sector. In this regard, an effective control measure such as immunization is very important due to the fact that it is cheaper than chemotherapy. In order to introduce effective immunization programs and ensure desirable protection of the local cattle, appropriately matched vaccine strains of *T. parva* need to be identified for use in new areas such as Zambia’s Chongwe and Chisamba districts through the application of molecular-based studies on prevailing *T. parva* populations. This is because the infection and treatment method only provides strain-specific immunity [16,27,28,29,30]. Such studies have recently produced positive results in Rwanda [8] with regards to the use of Muguga cocktails in field trials. Therefore, for the purpose of assessing the similarities between the Chongwe and Chisamba district field samples and the available vaccine stocks, this study detected and sequenced the Tp1 and Tp2 antigens of *T. parva* in cattle and characterized *T. parva* populations using satellite analysis. This is the first study that has utilized the sequence diversity of *T. parva* CTL antigens Tp1 and Tp2 and mini- and micro-satellite analysis to characterize and compare field samples with known vaccine isolates in Zambia.

Phylogenetic analysis of CTL Tp1 (Figure 1) and Tp2 (Figure 3) antigens from Chongwe and Chisamba districts showed six and three clusters, respectively, in comparison and contrast to other studies [8,9,10,21,22,31]. In Chongwe, the majority of samples were closely related to Muguga cocktail and Katete vaccine stocks, while the minority were similar to Chitongo. On the other hand, an almost equal distribution of similarity between Muguga cocktail and Katete strains and Chitongo was observed (Figure 1 and Figure 3). The implication of this is that most of the *T. parva* field population in Chongwe and Chisamba is highly similar to the Muguga cocktail and Katete vaccine strains as compared to Chitongo, and the few samples that did not show close similarity to any of the vaccine groups could represent a different minority population. This observation is in agreement with previous studies in different regions [8,10]. In both districts, evidence of expanding populations [22] with similar haplotypes across the different veterinary camps as well as negative purifying selection implied that the practice of open free-range grazing coupled with free trade in animals that is widely practised in both districts among the local farmers could be the main driver in spreading *T. parva* infection in different areas. This practice also prevents the implementation of adequate control strategies such as frequent dipping and livestock movement ban, which are important in mitigating the effects of theileriosis. The free movement of animals and absence of immunization campaigns at the time of sampling could have also led to some extent of enzootic stability, as evidenced by the negative purifying selection obtained in this study. However, sequence analysis alone is not enough to ascertain which vaccine strain is the most appropriate for use in field challenge trials with respect to *T. parva*. Further, owing to the highly conserved nature of Tp1 and diversity of Tp2 genes [8,9,10,21,22], it is difficult to determine with certainty the similarity of proposed vaccine isolates to the field populations. Thus, in order to improve the resolution of the results of sequence diversity, population genetic analysis encompassing nine (9) sites on the *T. parva* genome was performed. Population genetic analysis revealed high gene diversities in all populations across all loci (Table 2), implying the presence of diverse populations. Even though field samples from both districts shared similar epitopes with MC and Katete vaccine isolates on sequence analysis, they did not share any alleles with either on microsatellite analysis; instead, both shared a total of seven alleles with the Chitongo population (Figure 5d). This indicated that even though the field populations might be similar to the MC and Katete vaccine stocks immunologically, they are likely to be a different population from MC and Katete. The presence of the samples that shared alleles with Chitongo could represent a minor Chitongo population in both districts or could have been sampled from animals that had recently been introduced in both districts from the Southern province of Zambia, where immunization with Chitongo vaccine is practiced. The latter is more likely as it is well known that local farmers illegally move animals from the Southern province to other districts, Chongwe and Chisamba inclusive. When the Chongwe population was exclusively compared to vaccine isolates, Katete showed the highest similarity to the field populations (Figure 6e), implying that the most dominant population in this district was similar to the Katete isolate which is prevalent in the eastern part of Zambia. In comparison, the Chisamba population showed an almost equal distribution with regards to the similarity between the Chitongo and Katete vaccine isolates (Figure 6f), indicating that only Katete and Chitongo populations could have been prevailing and the earlier similarities observed on sequence diversity were likely towards Katete isolate as none of the samples showed any similarity to MC population (Figure 6f). In both districts, low genetic differentiation and a lack of evidence of clustering according to the origin of the sample was observed (Table 3). Despite the low genetic differentiation among the veterinary camps in both districts, LE and panmixia were absent (Table 3). This could possibly be attributed to the fact that most animals sampled were exhibiting clinical signs such as swollen lymph nodes and pyrexia and might not have been able to move around widely. Furthermore, the lack of high intensity of infection as a result of a mono-modal cycle of infection could have contributed to the absence of LE in contrast to previous studies [10]. When Chongwe and Chisamba were considered as one population, moderate genetic differentiation (Table 3) was observed despite the distance between the two districts. This could be attributed to the ease of movement of animals during grazing or trade between the two districts, as earlier highlighted. The level of population differentiation as evidenced by the F_ST_ value obtained in this study compared to the previous study [32] is lower and can be attributed to the absence of geographical barriers between the Chongwe and Chisamba districts. However, LD and non-random mating observed between the two districts also indicates that even though animals might move from one district to the other, this practice is probably not extensive enough to allow the free mixing of parasite populations and contribute to a situation of high intensity of infection.

Based on the sequence diversity and population genetic data, the most prevalent population causing disease in Chongwe district could be highly similar to Katete, while in Chisamba, two populations might be circulating, with Katete being the dominant and Chitongo being the minority. Katete stock is thus favored as a vaccine candidate in both districts because, among the vaccine isolates, it has the closest immunological profile to the field samples and is most prevalent in both districts. With regards to MC, previous attempts in the past did not yield desirable results and is not in use in Zambia [4]. Furthermore, MC is not as prevalent in the study areas as Katete and Chitongo and owing to the fact that Katete is in use as a vaccine in the eastern province of Zambia and has produced desirable results, it is a better candidate for field challenge trials in the study areas as this will minimize the introduction of new strains or populations in the study areas ultimately resulting in further loss of naive livestock. Therefore, it can be hypothesized that field challenge trials using Katete vaccine isolate in both districts might produce desirable levels of protection in local animals. Through a similar analytical approach as this study, ITM using MC has been attempted in regions such as Rwanda with desirable results [8].

In conclusion, this study has shown that the most prevalent *T. parva* population circulating in Chongwe and Chisamba districts was similar to the Katete vaccine population, and between the two districts, moderate genetic differentiation and geographical sub-structuring was observed. Furthermore, a field challenge trial using the Katete vaccine strain is likely to produce a stronger immune response and protection against theileriosis in Chongwe and Chisamba districts.

## 4. Materials and Methods

### 4.1. Study Sites

**Figure 7:**
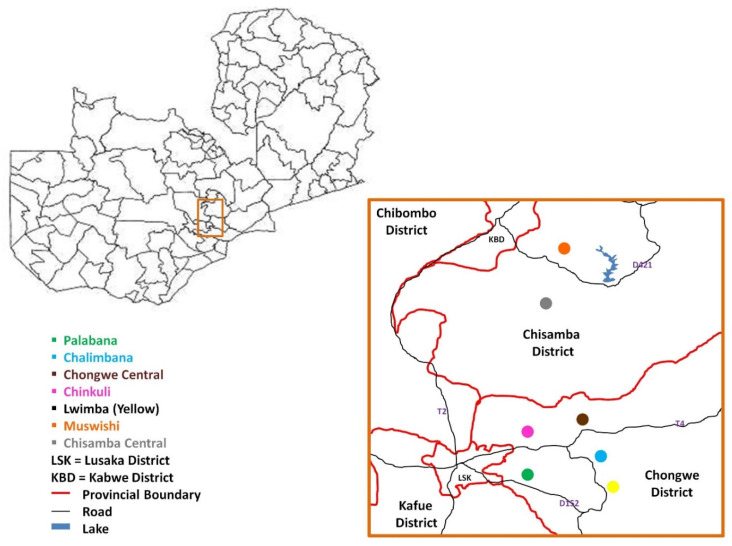
Map of Zambia showing its districts and veterinary camps from where samples were obtained. Veterinary camps from Chongwe are; Palabana (green), Chalimbana (blue), Chongwe central (Brown), Chinkuli (pink) and Lwimba (yellow). Veterinary camps sampled from Chisamba district are Muswishi (orange) and Chisamba central (grey).

Chongwe district lies approximately 50 km east of Lusaka town within Lusaka province, and Chisamba district, in the Central province, is approximately 60 km north of Lusaka town. The study sites in Chongwe district were Lwimba, Chongwe Central, Chikuli, Palabana and Chalimbana veterinary camps, while those for Chisamba district were Chisamba central and Muswishi veterinary camps (Figure 7). In both districts, the majority of cattle are kept under free-range grazing.

### 4.2. Sample Collection and DNA Extraction

Whole blood was collected in EDTA vacutainer tubes from cattle in Chongwe (*n* = 130) and Chisamba (*n* = 29) districts in February 2017. In both districts, samples were collected from unvaccinated animals presenting with swollen lymph nodes and pyrexia. No previous vaccination programs had been implemented in either district prior to sampling. As such, these herds of cattle were considered naive. Extraction of genomic DNA from whole blood samples was achieved using a commercial extraction kit (Thermo Scientific, Santa Clara, CA, USA) based on the manufacturer’s instructions.

### 4.3. PCR Screening of Theileria parva

*Theileria parva* specific primers [33] targeting the p104 gene were used to screen samples for the presence of *T. parva* genome. Briefly, the p104 gene was amplified from template DNA using the amplitaq gold PCR kit. The reaction mix was as described by the manufacturer, and the PCR cycles were as follows; denaturation at 95 °C for 10 min followed by 35 cycles of 95 °C for 60 s, 63 °C for 30 s and 68 °C for 1 min and a final extension step of 68 °C for 5 min. The PCR products were visualized on 1.5% agarose gel stained with ethidium bromide.

### 4.4. PCR Amplification of Tp1 and Tp2 Genes

*Theileria parva* Tp1 and Tp2 genes were amplified using Amplitaq Gold PCR kit (Invitrogen, Carlsbad, CA, USA) according to the manufacturer’s instructions using primers previously described [21]. The PCR protocol and cycles included; 95 °C denaturation for 10 min followed by 35 cycles at 96 °C for 30 s, annealing at 50 °C (Tp1) and 53 °C (Tp2) for 45 s and extension at 68 °C for 60 s. A final extension of 5 min at 68 °C was also incorporated. PCR amplification products were visualized on 2% agarose gel stained with ethidium bromide.

### 4.5. Cycle Sequencing

PCR products were prepared for cycle sequencing by purifying the excess buffers and dNTPs using the Monofas purification kit (GL Sciences, Tokyo, Japan) according to the manufacturer’s instructions. The purified products were then subjected to cycle sequencing PCR using the Big Dye Terminator v3.1 cycle sequencing kit (Life Technologies, Applied Biosystems, CA, USA). Following successful cycle sequencing PCR, the ethanol precipitation method was used to remove the unincorporated labeled dNTPs, buffers and enzymes. The resultant purified sequence products were then denatured and subjected to capillary electrophoresis on the ABI 3500 genetic analyzer (Life Technologies).

### 4.6. Microsatellite PCR

*Theileria parva* positive field samples, Muguga, Kiambu, Serengeti, Katete and Chitongo vaccine isolates were genotyped using a panel of nine (9) markers (Table 4). From each primer pair, the forward primer was fluorescently labeled. Amplitaq Gold master mix PCR kit (Invitrogen) was used to amplify the target regions according to the manufacturer’s instructions. The PCR conditions were denaturation at 95 °C for 10 min followed by 35 cycles of 96 °C for 1 min, annealing for 30 s and 68 °C for 1 min with a final extension of 72 °C for 5 min. Annealing temperatures for each primer pair are given in Table 4. The success of PCR amplification was verified using 2% agarose gel coated with ethidium bromide. The PCR products were then denatured and electrophoresed using the ABI Seqstudio genetic analyzer (Life Technologies). The DNA fragment sizes obtained were determined using the GeneMapper software ver. 5 (Applied Biosystem, Waltham, MA, USA), which scored peaks with the highest area as the most dominant allele. Finally, a multi-locus genotype (MLG) to represent the dominant genotype within each sample was then constructed using DNA fragment sizes.

## 5. Data Analysis

### 5.1. Sequence Analysis

All the nucleotide sequences obtained were subjected to blast analysis on the NCBI website and then edited and assembled using the ATGC software plug-in in Genetyx ver. 12 (Genetyx Corporation, Tokyo, Japan). Clustal W1.6 was used to generate multiple sequence alignments of the obtained sequences and the reference sequences. Among the reference sequences used were Muguga, Kiambu and Serengeti transformed. Amino acid sequences were then translated from the nucleotide sequence alignment file and used to determine the similarity of the epitopes between the samples and the Muguga cocktail, Katete and Chitongo vaccine stocks. Fasta files of the multiple sequence alignments for Tp1 and Tp2 genes were then converted to a mega file and then used to generate neighbor-joining phylogenetic trees with a confidence level of 1000 bootstrap replicates using Mega ver. 6 computer software [34]. DNA nucleotide polymorphisms, that is, the average number of nucleotide differences per site for both Tp1 and Tp2 in the samples was calculated using the computer program DnaSP ver. 5 [35]. Selection pressure on the Tp1 and Tp2 genes was assessed by determining the mean ratio of the non-synonymous substitutions and synonymous substitution (dN/dS) per site utilizing the F81 model with a 0.05 confidence level incorporated in the single likelihood ancestor counting (SLAC) method. The data was interpreted as follows, dN/dS = 1, <1 and >1 indicates neutrality, negative selection and positive selection, respectively. All analyses were performed using the Datamonkey website interface (Available online: http://www.datamonkey.org, accessed on 8 July 2021) [36,37].

### 5.2. Haplotype Similarity Analysis

The similarities between the Tp1 and Tp2 haplotypes in Muguga, Kiambu, Serengeti, Chitongo, Katete vaccine isolates and Zambian samples were assessed by constructing a median joining (MJ) network link using Network 4.5 computer software (https://fluxus-engineering.com/sharenet.htm, accessed on 26 July 2021 [26]. All nucleotide sequences obtained in this study were deposited in the DNA Data Bank of Japan (DDBJ) with accession numbers LC645702-LC645968 (Supplementary Table S2).

### 5.3. Microsatellite Analysis

Within the MLG, the level of similarity was analyzed using the microsatellite tool kit (http://animalgenomics.ucd.i.e.,/sdepark/ms-toolkit/, accessed on 26 July 2021) and then visualized using the allele frequency distribution and principal component analysis (PCA) constructed using GenAIEx6 [38]. Further, AMOVA and the extent of population differentiation was calculated using the Arlequin computer package version 3.5 [39], and the null hypothesis of panmixia (random mating) and linkage equilibrium was assessed using LIAN [40], computer software that calculates the variance of pairwise differences (V_D_), the standardized index of association, the variance of differences required for panmixia (V_E_) and the 95% confidence interval (L) for V_D_. LIAN data output was interpreted as follows; when values that were negative or close to zero were obtained for the standard index of association, panmixia (random mating) was indicated, while positive values or those significantly greater than zero indicated non-panmixia (non-random mating). Furthermore, when the calculated V_D_ value was less than L, linkage equilibrium (LE) was indicated and the null hypothesis of panmixia was accepted, and when V_D_ was greater than L, linkage disequilibrium (LD) was indicated and the null hypothesis of panmixia was rejected.

## Figures and Tables

**Table 1:** Tp2 epitopes identified in unvaccinated cattle from Chongwe and Chisamba districts of Zambia that are different from Muguga cocktail epitopes.

	CTL 1	CTL 2	CTL 3	CTL 4	CTL 5	CTL 6
	SHEEL**N**KLGML (9)	**PDL**D**KNT**LF (9)	**LT**SHGMGK**I**G**R** (3)	**L**A**P**S**IK**CV**S** (2)	**A**S**IK**CV**AQ**K (1)	K**P**S**V**PNPC**I**W (9)
	S**D**EEL**DN**LGM**F** (1)	**H**G**L**D**KNT**LF (1)	**L**SSHGMG**RI**G**R** (8)	**L**A**A**S**IK**CV**S** (1)	**P**S**IK**CV**SHH** (2)	KTSIPNPC**E**W (1)
	S**D**EEL**DN**LGML (1)	**PDL**DR**NT**LF (3)	**L**SSHGMG**RI**GK (5)	FA**A**S**IK**CV**S** (2)	**A**S**IK**CV**SHH** (1)	K**N**SIPNPCKW (1)
	S**DD**EL**DN**LGML (4)	D**DL**DR**N**ALF (1)	**L**SSHGMGKVGK (3)	FA**P**S**IK**CV**A** (1)	**A**S**IK**CV**SQY** (2)	K**P**S**V**PNPCKW (2)
	SHEELKK**M**GM**V** (1)	**HD**FD**KNT**LF (1)	**LP**SHGMG**RI**G**R** (2)	FAQSL**M**CV**S** (1)	**P**S**IK**CV**AQY** (1)	KT**D**IPNPCKW (2)
	S**D**E**G**L**N**KLGML (1)	**QDL**D**KNT**LF (1)	**R**SSHGMGKVGK (1)	FAQSL**M**CVL (10)	QSL**M**CV**SQ**K (1)	K**P**SIPNPCKW (1)
	S**DD**EL**NN**LGML (4)	**PDS**DR**NT**LF (1)	**L**SSHGMGK**I**GK (2)	FA**P**SL**M**CVL (1)	QSL**M**CVL**Q**K (1)	K**P**S**G**PNPC**I**W (1)
	S**DN**EL**DT**LG**L**L (1)	**HD**FDR**NT**LF (1)	LSSHGMGK**I**GK (1)	FA**A**SL**M**CV**S** (1)	QSL**M**CVL**L**K (1)	**V**T**D**IPNPCKW (1)
	S**D**EEL**DT**LGML (1)	**E**GFD**K**DALF (1)	**I**SSHGMGKVGK (1)	FAQSL**K**CVL (1)	**P**SL**M**CVL**LN** (1)	
	S**DN**EL**DT**LG**L**L (1)	**H**G**LA**R**NT**LF (1)	KSS**QS**MG**I**VG**R** (1)	FA**A**S**IK**CVL (2)	**A**SL**M**CV**SL**K (1)	
	S**ED**EL**DT**LGML (1)	DG**L**DR**NT**LF (1)		F**V**QS**IM**CV**I** (1)	QSL**M**CVLMK (9)	
	S**DD**EL**N**KLGML (2)	DG**L**DRDALF (1)			QSL**K**CVL**L**K (1)	
	S**DD**ELK**N**LG**L**L (1)	D**D**FDRDALF (1)			**A**S**IK**CVL**QY** (2)	
	S**DN**EL**DT**LG**L**L (1)	DGFDR**N**ALF (1)			QSLVCVL**L**K (1)	
	S**D**EEL**N**K**S**GML (1)	D**D**FDRD**T**LF (1)			QS**IM**CV**IN**K (1)	
	**TE**EEL**NNM**GM**V** (1)	**E**GFD**K**DALF (1)				
**Total**	**31**	**26**	**27**	**24**	**26**	**18**

**Table 2:** Allelic variation among *Theileria parva* populations from Chongwe and Chisamba districts of Zambia.

	Population	N	MS2	MS7	ms9	MS14	MS15	MS19	MS25	MS33	MS39
Alleles within the Populations	Chongwe	96	13	23	17	55	14	27	17	40	21
	Chisamba	29	8	14	4	6	5	6	5	14	4
	Muguga cocktail	3	1	1	1	1	1	2	1	1	1
	Katete	2	2	1	2	2	2	2	2	2	2
	Chitongo	2	1	2	1	2	1	1	2	2	1
Gene Diversity	Chongwe	96	0.856	0.907	0.784	0.970	0.708	0.942	0.878	0.953	0.894
	Chisamba	29	0.833	0.897	0.554	0.374	0.613	0.719	0.495	0.921	0.707
	Muguga cocktail	3	0.00	0.00	0.00	0.00	0.00	0.667	0.00	0.00	0.00
	Katete	2	1.00	0.00	1.00	1.00	1.00	1.00	1.00	1.00	1.00
	Chitongo	2	0.00	1.00	0.00	1.00	0.00	0.00	1.00	1.00	0.00
	Overall	132	0.538	0.561	0.468	0.669	0.464	0.666	0.675	0.775	0.520

**Table 3:** Population genetic analyses of *Theileria parva* from Chongwe and Chisamba districts of Zambia.

Population	N	Effective Alleles	Ht	VD	L	*p*-Value	ISA	Linkage	FST
Chongwe	96	6.086	0.8767	1.1059	0.9673	<0.01	0.0260	LD	0.036
Chisamba	29	3.574	0.6719	2.0003	1.8916	<0.01	0.0233	LD	−0.012
Chongwe + Chisamba	125	7.426	0.8629	1.3044	1.0620	<0.01	0.0373	LD	0.0960
Chongwe + Chisamba + Vaccines	132	3.855	0.8641	1.3357	1.0518	<0.01	0.0421	LD	0.1205

N: number of samples, H_t_: Estimated Heterozygosity, V_D_: mismatch variance (linkage analysis), L: upper 95% confidence limit of Monte Carlo simulation (linkage analysis), ISA: Standard Index of Association.

**Table 4:** Panel of microsatellite markers used to genotype *Theileria parva* samples from Chongwe and Chisamba districts, Zambia.

Marker	Chromosome	Colour	Annealing Temp	Bp
MS2	1	Red	60	342–562
MS3	1	Red	60	193–380
MS8	1	Red	60	160–330
MS14	2	Yellow	60	360–600
MS15	2	Red	60	120–220
MS21	3	Red	60	170–400
MS27	3	Yellow	60	130–220
MS30	3	Red	60	170–230
MS33	4	Yellow	60	150–220
MS40	4	Red	60	150–250
MS25	3	Red	50	324
MS39	4	Yellow	50	263
MS7	1	Red	50	372
MS19	2	Yellow	50	304
ms9	3	Yellow	50	230

## Data Availability

All the nucleotide sequences obtained and used in this study have been deposited in the DNA database of Japan with accession numbers LC645702-LC645968. All other material is contained within the manuscript.

## Supplementary Materials

The following are available online at https://www.mdpi.com/article/10.3390/pathogens11020114/s1, Table S1: Summary of sequences that share 100% identity on Tp1 and Tp2.title; Table S2: List of samples and accession numbers used in this study.
