# Supplementary material for: Sequence Diversity of Tp1 and Tp2 Antigens and Population Genetic Analysis of Theileria parva in Unvaccinated Cattle in Zambia’s Chongwe and Chisamba Districts
Source: Pathogens. 2022 Jan 19;11(2):114. doi: 10.3390/pathogens11020114 (PMC8879479; doi:10.3390/pathogens11020114)
Supplement: Supplementary file 1 [file pathogens-11-00114-s001.zip › pathogens-1524737-Table S1.pdf]

Supplementary Table S1. Summary of sequences that share 100% identity on Tp1 and Tp2.

Gene	Group	Representative Sample	Number in group	Samples in each group
**Tp1**	1	8C7 Chalimbana	25	5X8 Chalimbana, 9X9 Chalimbana, 11C8 Chalimbana, 16X11 Chalimbana, 18X12 Chalimbana, 25X14 Chalimbana 29X15 Chalimbana, 31X62 Chalimbana, 35X16 Chalimbana, 40X63 Chalimbana, 41C23 Chalimbana, 44X17 Chalimbana, 48X18 Chalimbana, 50C27 Chalimbana, 56X64 Chinkuli, 59C34 Chinkuli, 104X48 Chinkuli, 62C37 Lwimba, 67C42 Lwimba, 73X22 Palabana, 91C60 Chongwe Central, 92C61 Chongwe Central, 94X23 Chongwe Central, 99C68 Chongwe Central
	2	72C47 Palabana	4	06X60 Chalimbana, 10X10 Chalimbana, 117X57 Chinkuli
	3	15C11 Chalimbana	18	19C13 Chalimbana, 21C14 Chalimbana, 22C15 Chalimbana, 23C16 Chalimbana, 28C17 Chalimbana, 31C18 Chalimbana, 36C20 Chalimbana, 38C21 Chalimbana, 42C24 Chalimbana, 48C26 Chalimbana, 12C19 Chalimbana, 53X65 Chinkuli, 54C30 Chinkuli, 60C35 Chinkuli, 119X58 Chinkuli, 97C66 Chongwe Central, 98C67 Chongwe Central
	4	3C3 Challimbana	3	105X49 Chinkuli, 74C49 Palabana
	5	58C29 Lwimba	10	55C31 Chinkuli, 116X67 Chinkuli, 56C32 Lwimba, 63C38 Lwimba, 64C39 Lwimba, 66X19 Lwimba, 72C55 Palabana, 86C54 Chongwe Central, 87C56 Chongwe Central
	6	70C45 Lwimba	6	100C69 Chinkuli, 71C46 Palabana, 73C48 Palabana, 79C52 Chongwe Central, 82C53 Chongwe Central
**Tp2**	1	5C2 Chalimbana	4	11C6 Chalimbana, 56C34 Chinkuli, 65C42 Chinkuli
	2	63C40 Lwimba	52	23C14 Chalimbana, 28C16 Chalimbana, 29C17 Chalimbana, 31C18 Chalimbana, 32C19 Chalimbana, 64C41 Lwimba, 69C45 Lwimba, 81C52 Chongwe Central, 96C64 Chongwe Central, 2X21 Chalimbana, 3X22 Chalimbana, 6X76 Chalimbana, 12X40 Chalimbana, 16X26 Chalimbana, 16X41

				Chalimbana, 17X42 Chalimbana, 21X43 Chalimbana, 35X27 Chalimbana, 40X44 Chalimbana, 44X28 Chalimbana, 48X45 Chalimbana, 51X29 Lwimba, 56X46 Lwimba, 58X30 Chinkuli, 64X32 Lwimba, 65X33 Chinkuli, 68X34 Lwimba, 68X47 Lwimba, 70X35 Lwimba, 71X48 Palabana, 72x49 Palabana, 73X36 Palabana, 73x50 Palabana, 76X38 Palabana, 80X52 Chongwe Central, 82X53 Chongwe Central, 83X54 Chongwe Central, 88X55 Chongwe Central, 90X56 Chongwe Central, 94X57 Chongwe Central, 95X39 Chongwe Central, 96X58 Chongwe Central, 97X59 Chongwe Central, 100X60 Chinkuli, 103X61 Chinkuli, 104X62 Chinkuli, 105X64 Chinkuli, 111X68 Chinkuli, 116X63 Chinkuli, 121X74 Chinkuli, 122X75 Chinkuli
	3	50C29 Chinkuli	13	36C22 Chalimbana, 61C38 Lwimba, 76C49 Palabana, 88C56 Chongwe Central, 94C62 Chongwe Central, 9X23 Chalimbana, 14X25 Chalimbana, 53X80 Chinkuli, 108X65 Chinkuli, 110X67 Chinkuli, 118X72 Chinkuli, 127X81 Chinkuli
	4	78C50 Chongwe Central	4	91C59 Chongwe Central, 99C67 Chongwe Central, 79x51 Chongwe Central
	5	74X37 Palabana	2	31X78 Chalimbana
