# Supplementary material for: Sequence Diversity of Tp1 and Tp2 Antigens and Population Genetic Analysis of Theileria parva in Unvaccinated Cattle in Zambia’s Chongwe and Chisamba Districts
Source: Pathogens. 2022 Jan 19;11(2):114. doi: 10.3390/pathogens11020114 (PMC8879479; doi:10.3390/pathogens11020114)
Supplement: Supplementary file 1 [file pathogens-11-00114-s001.zip › pathogens-1524737-Table S2.pdf]

Supplementary Table S2. List of samples and accession numbers used in this study

Gene	Sample ID	District	Veterinary Camp	Accession number
Tp2	4C1	Chongwe	Chalimbana	LC645702
	5C2	Chongwe	Chalimbana	LC645703
	6C3	Chongwe	Chalimbana	LC645704
	7C4	Chongwe	Chalimbana	LC645705
	8C5	Chongwe	Chalimbana	LC645706
	11C6	Chongwe	Chalimbana	LC645707
	12C7	Chongwe	Chalimbana	LC645708
	13C8	Chongwe	Chalimbana	LC645709
	19C10	Chongwe	Chalimbana	LC645710
	20C11	Chongwe	Chalimbana	LC645711
	21C12	Chongwe	Chalimbana	LC645712
	22C13	Chongwe	Chalimbana	LC645713
	23C14	Chongwe	Chalimbana	LC645714
	24C15	Chongwe	Chalimbana	LC645715
	28C16	Chongwe	Chalimbana	LC645716
	29C17	Chongwe	Chalimbana	LC645717
	31C18	Chongwe	Chalimbana	LC645718
	32C19	Chongwe	Chalimbana	LC645719
	33C20	Chongwe	Chalimbana	LC645720
	35C21	Chongwe	Chalimbana	LC645721
	36C22	Chongwe	Chalimbana	LC645722
	38C23	Chongwe	Chalimbana	LC645723
	40C24	Chongwe	Chalimbana	LC645724
	41C25	Chongwe	Chalimbana	LC645725
	42C26	Chongwe	Chalimbana	LC645726
	45C27	Chongwe	Chalimbana	LC645727
	48C28	Chongwe	Chalimbana	LC645728
	50C29	Chongwe	Chinkuli	LC645729
	51C30	Chongwe	Chinkuli	LC645730
	52C31	Chongwe	Chinkuli	LC645731
	54C32	Chongwe	Chinkuli	LC645732
	55C33	Chongwe	Chinkuli	LC645733
	56C34	Chongwe	Chinkuli	LC645734
	57C35	Chongwe	Chinkuli	LC645735
	58C36	Chongwe	Chinkuli	LC645736
	59C37	Chongwe	Lwimba	LC645737
	61C38	Chongwe	Lwimba	LC645738
	62C39	Chongwe	Lwimba	LC645739
	63C40	Chongwe	Lwimba	LC645740
	64C41	Chongwe	Lwimba	LC645741
	65C42	Chongwe	Chinkuli	LC645742
	66C43	Chongwe	Palabana	LC645743

	68C44	Chongwe	Lwimba	LC645744
	69C45	Chongwe	Lwimba	LC645745
	70C46	Chongwe	Lwimba	LC645746
	72C47	Chongwe	Palabana	LC645747
	75C48	Chongwe	Palabana	LC645748
	76C49	Chongwe	Palabana	LC645749
	78C50	Chongwe	Chongwe Central	LC645750
	80C51	Chongwe	Chongwe Central	LC645751
	81C52	Chongwe	Chongwe Central	LC645752
	86C54	Chongwe	Chongwe Central	LC645753
	87C55	Chongwe	Chongwe Central	LC645754
	88C56	Chongwe	Chongwe Central	LC645755
	89C57	Chongwe	Chongwe Central	LC645756
	90C58	Chongwe	Chongwe Central	LC645757
	91C59	Chongwe	Chongwe Central	LC645758
	92C60	Chongwe	Chongwe Central	LC645759
	93C61	Chongwe	Chongwe Central	LC645760
	94C62	Chongwe	Chongwe Central	LC645761
	95C63	Chongwe	Chongwe Central	LC645762
	96C64	Chongwe	Chongwe Central	LC645763
	97C65	Chongwe	Chongwe Central	LC645764
	98C66	Chongwe	Chongwe Central	LC645765
	99C67	Chongwe	Chongwe Central	LC645766
	100C68	Chongwe	Chinkuli	LC645767
	101C69	Chongwe	Chinkuli	LC645768
	102C70	Chongwe	Chinkuli	LC645769
	1X20	Chongwe	Chalimbana	LC645770
	1X21	Chongwe	Chalimbana	LC645771
	3X22	Chongwe	Chalimbana	LC645772
	6X76	Chongwe	Chalimbana	LC645773
	9X23	Chongwe	Chalimbana	LC645774
	10X24	Chongwe	Chalimbana	LC645775
	12X40	Chongwe	Chalimbana	LC645776
	14X25	Chongwe	Chalimbana	LC645777
	16X26	Chongwe	Chalimbana	LC645778
	16X41	Chongwe	Chalimbana	LC645779
	17X42	Chongwe	Chalimbana	LC645780
	21X43	Chongwe	Chalimbana	LC645781
	31X78	Chongwe	Chalimbana	LC645782
	35X27	Chongwe	Chalimbana	LC645783
	40X44	Chongwe	Chalimbana	LC645784
	40X79	Chongwe	Chalimbana	LC645785
	44X28	Chongwe	Chalimbana	LC645786
	48X45	Chongwe	Chalimbana	LC645787
	51X29	Chongwe	Lwimba	LC645788

	53X80	Chongwe	Chinkuli	LC645789
	56X46	Chongwe	Lwimba	LC645790
	58X30	Chongwe	Chinkuli	LC645791
	62X31	Chongwe	Lwimba	LC645792
	64X32	Chongwe	Lwimba	LC645793
	65X33	Chongwe	Chinkuli	LC645794
	68X34	Chongwe	Lwimba	LC645795
	68X47	Chongwe	Lwimba	LC645796
	70X35	Chongwe	Lwimba	LC645797
	71X48	Chongwe	Palabana	LC645798
	72X49	Chongwe	Palabana	LC645799
	73X36	Chongwe	Palabana	LC645800
	73X50	Chongwe	Palabana	LC645801
	74X37	Chongwe	Palabana	LC645802
	76X38	Chongwe	Palabana	LC645803
	79X51	Chongwe	Chongwe Central	LC645804
	80X52	Chongwe	Chongwe Central	LC645805
	82X53	Chongwe	Chongwe Central	LC645806
	83X54	Chongwe	Chongwe Central	LC645807
	88X55	Chongwe	Chongwe Central	LC645808
	90X56	Chongwe	Chongwe Central	LC645809
	94X57	Chongwe	Chongwe Central	LC645810
	95X39	Chongwe	Chongwe Central	LC645811
	96X58	Chongwe	Chongwe Central	LC645812
	97X59	Chongwe	Chongwe Central	LC645813
	100X60	Chongwe	Chinkuli	LC645814
	103X61	Chongwe	Chinkuli	LC645815
	104X62	Chongwe	Chinkuli	LC645816
	105X64	Chongwe	Chinkuli	LC645817
	108X65	Chongwe	Chinkuli	LC645818
	109X66	Chongwe	Chinkuli	LC645819
	110X67	Chongwe	Chinkuli	LC645820
	111X68	Chongwe	Chinkuli	LC645821
	114X69	Chongwe	Chinkuli	LC645822
	115X70	Chongwe	Chinkuli	LC645823
	116X63	Chongwe	Chinkuli	LC645824
	118X72	Chongwe	Chinkuli	LC645825
	120X73	Chongwe	Chinkuli	LC645826
	121X74	Chongwe	Chinkuli	LC645827
	122X75	Chongwe	Chinkuli	LC645828
	127X81	Chongwe	Chinkuli	LC645829
	2BA55	Chisamba	Muswishi	LC645830
	3BA56	Chisamba	Muswishi	LC645831
	5BA57	Chisamba	Muswishi	LC645832
	6BA58	Chisamba	Muswishi	LC645833

	9BA59	Chisamba	Muswishi	LC645834
	13BA60	Chisamba	Chisamba Central	LC645835
	15BA61	Chisamba	Chisamba Central	LC645836
	16BA62	Chisamba	Chisamba Central	LC645837
	20BA63	Chisamba	Chisamba Central	LC645838
	22BA64	Chisamba	Chisamba Central	LC645839
	24BA65	Chisamba	Chisamba Central	LC645840
	25BA66	Chisamba	Chisamba Central	LC645841
	26BA69	Chisamba	Chisamba Central	LC645842
	27BA67	Chisamba	Chisamba Central	LC645843
Tp1	KL 11	Katete	Katete	LC645844
	5X8	Chongwe	Chalimbana	LC645845
	06X60	Chongwe	Chalimbana	LC645846
	9X9	Chongwe	Chalimbana	LC645847
	10X10	Chongwe	Chalimbana	LC645848
	11C8	Chongwe	Chalimbana	LC645849
	12C9	Chongwe	Chalimbana	LC645850
	16X11	Chongwe	Chalimbana	LC645851
	18X12	Chongwe	Chalimbana	LC645852
	19C13	Chongwe	Chalimbana	LC645853
	21C14	Chongwe	Chalimbana	LC645854
	22C15	Chongwe	Chalimbana	LC645855
	23C16	Chongwe	Chalimbana	LC645856
	25X14	Chongwe	Chalimbana	LC645857
	28C17	Chongwe	Chalimbana	LC645858
	29X15	Chongwe	Chalimbana	LC645859
	31C18	Chongwe	Chalimbana	LC645860
	31X62	Chongwe	Chalimbana	LC645861
	35X16	Chongwe	Chalimbana	LC645862
	36C20	Chongwe	Chalimbana	LC645863
	38C21	Chongwe	Chalimbana	LC645864
	40X63	Chongwe	Chalimbana	LC645865
	41C23	Chongwe	Chalimbana	LC645866
	42C24	Chongwe	Chalimbana	LC645867
	44X17	Chongwe	Chalimbana	LC645868
	48C26	Chongwe	Chalimbana	LC645869
	48X18	Chongwe	Chalimbana	LC645870
	50C27	Chongwe	Chalimbana	LC645871
	1C1	Chongwe	Chalimbana	LC645872
	2C2	Chongwe	Chalimbana	LC645873
	3C3	Chongwe	Chalimbana	LC645874
	4C4c	Chongwe	Chalimbana	LC645875
	6C5	Chongwe	Chalimbana	LC645876
	7C6	Chongwe	Chalimbana	LC645877

	8C7	Chongwe	Chalimbana	LC645878
	12C19	Chongwe	Chalimbana	LC645879
	13C10	Chongwe	Chalimbana	LC645880
	15C11	Chongwe	Chalimbana	LC645881
	17C12	Chongwe	Chalimbana	LC645882
	45C25	Chongwe	Chalimbana	LC645883
	53X65	Chongwe	Chinkuli	LC645884
	54C30	Chongwe	Chinkuli	LC645885
	55C31	Chongwe	Chinkuli	LC645886
	56X64	Chongwe	Chinkuli	LC645887
	59C34	Chongwe	Chinkuli	LC645888
	60C35	Chongwe	Chinkuli	LC645889
	100C69	Chongwe	Chinkuli	LC645890
	104X48	Chongwe	Chinkuli	LC645891
	105X49	Chongwe	Chinkuli	LC645892
	116X67	Chongwe	Chinkuli	LC645893
	117X57	Chongwe	Chinkuli	LC645894
	119X58	Chongwe	Chinkuli	LC645895
	58C33	Chongwe	Chinkuli	LC645896
	65C40	Chongwe	Chinkuli	LC645897
	101C70	Chongwe	Chinkuli	LC645898
	107X50	Chongwe	Chinkuli	LC645899
	108X51	Chongwe	Chinkuli	LC645900
	109X52	Chongwe	Chinkuli	LC645901
	110X53	Chongwe	Chinkuli	LC645902
	116X56	Chongwe	Chinkuli	LC645903
	122X59	Chongwe	Chinkuli	LC645904
	56C32	Chongwe	Lwimba	LC645905
	62C37	Chongwe	Lwimba	LC645906
	63C38	Chongwe	Lwimba	LC645907
	64C39	Chongwe	Lwimba	LC645908
	66X19	Chongwe	Lwimba	LC645909
	51C28	Chongwe	Lwimba	LC645910
	58C29	Chongwe	Lwimba	LC645911
	61C36	Chongwe	Lwimba	LC645912
	66C41	Chongwe	Lwimba	LC645913
	67X20	Chongwe	Lwimba	LC645914
	68C43	Chongwe	Lwimba	LC645915
	69C44	Chongwe	Lwimba	LC645917
	70C45	Chongwe	Lwimba	LC645918
	67C42	Chongwe	Lwimba	LC645919
	71X21	Chongwe	Palabana	LC645920
	71C46	Chongwe	Palabana	LC645921
	72C55	Chongwe	Palabana	LC645922
	73X22	Chongwe	Palabana	LC645923

	72C47	Chongwe	Palabana	LC645924
	77C50	Chongwe	Palabana	LC645925
	74C49	Chongwe	Palabana	LC645926
	79C52	Chongwe	Chongwe Central	LC645927
	82C53	Chongwe	Chongwe Central	LC645928
	86C54	Chongwe	Chongwe Central	LC645929
	87C56	Chongwe	Chongwe Central	LC645930
	91C60	Chongwe	Chongwe Central	LC645931
	92C61	Chongwe	Chongwe Central	LC645932
	94X23	Chongwe	Chongwe Central	LC645933
	97C66	Chongwe	Chongwe Central	LC645934
	98C67	Chongwe	Chongwe Central	LC645935
	99C68	Chongwe	Chongwe Central	LC645936
	78C51	Chongwe	Chongwe Central	LC645937
	88C57	Chongwe	Chongwe Central	LC645938
	89C58	Chongwe	Chongwe Central	LC645939
	90C59	Chongwe	Chongwe Central	LC645940
	94C63	Chongwe	Chongwe Central	LC645941
	1BA70	Chisamba	Muswishi	LC645942
	2BA71	Chisamba	Muswishi	LC645943
	3BA72	Chisamba	Muswishi	LC645944
	4BA73	Chisamba	Muswishi	LC645945
	5BA74	Chisamba	Muswishi	LC645946
	6BA75	Chisamba	Muswishi	LC645947
	7BA76	Chisamba	Muswishi	LC645948
	8BA77	Chisamba	Muswishi	LC645949
	09BA78	Chisamba	Muswishi	LC645950
	10BA79	Chisamba	Muswishi	LC645951
	11BA80	Chisamba	Muswishi	LC645952
	12BA81	Chisamba	Muswishi	LC645953
	13BA82	Chisamba	Chisamba Central	LC645954
	14BA83	Chisamba	Chisamba Central	LC645955
	15BA84	Chisamba	Chisamba Central	LC645956
	16BA85	Chisamba	Chisamba Central	LC645957
	17BA86	Chisamba	Chisamba Central	LC645958
	18BA87	Chisamba	Chisamba Central	LC645959
	19BA88	Chisamba	Chisamba Central	LC645960
	20BA89	Chisamba	Chisamba Central	LC645961
	21BA90	Chisamba	Chisamba Central	LC645962
	22BA91	Chisamba	Chisamba Central	LC645963
	23BA92	Chisamba	Chisamba Central	LC645964
	24BA93	Chisamba	Chisamba Central	LC645965
	25BA94	Chisamba	Chisamba Central	LC645966
	26BA95	Chisamba	Chisamba Central	LC645967
	27BA96	Chisamba	Chisamba Central	LC645968
